# Current News and Opinion

**Published:** 1883-02

**Authors:** 


### Current News and Opinion.

New York Pathological Society.—The following officers were elected at the annual meeting held on the evening of January 10: Dr. George F. Shrady, President; Dr. R. E. Van Gieson, Vice-President; Dr. Wesley M. Carpenter, Secretary; Dr. John H. Hinton, Treasurer, and Dr. John C. Peters, Editor of Transactions. Drs. F. R. S. Drake, J. H. Ripley, J. H. Hinton, V. P. Gibney, and J. C. Peters, Committee on Admissions and Ethics; Dr. J. C. Peters (Editor of Transactions), W. M. Carpenter, J. H. Hinton, E. C. Wendt, Beverley Robinson, and the President, Committee on Publication.

New York Academy of Medicine—Election of Officers.—At the annual meeting, held January 4, 1883, the following officers were elected: President, Fordyce Barker, M.D., LL.D.; Vice-President, H. P. Farnham, M.D.; Recording Secretary, W. H. Katzenbach, M.D.; Corresponding Secretary, J. G. Adams, M.D.; Treasurer, William F. Cushman, M.D.; Trustee, Gouverneur M. Smith, M.D.; Treasurer of Board of Trustees, Charles Wright, M.D.; Member of Committee on Admissions, E. L. Partridge, M.D.; Member of Committee on Ethics, H. E. Crampton, M.D.; Member of Committee on Education, J. C. Dalton, M.D.; Member of Committee on Library, A. McLane Hamilton, M.D.

The Twelfth Annual Dinner of the Alumni Association of the Medical Department of the New York University, was held at Delmonico's on Wednesday evening, January 24. About one hundred were present. Before the dinner, a business meeting was held, at which Dr. A. E. McDonald was elected President of the Association for the ensuing year. Toasts were responded to by Drs. Loomis, McDonald, A. A. Smith, of the Bellevue Hospital Medical College, Commissioner Brennan, Noah Brooks, Esq., and the Rev. Drs. Hall and Taylor. The evening was most enjoyable, all the speeches being of the shortest and wittiest, which, with the Delmonico *menu*, left nothing to be desired.

The Autopsy performed on the body of Gambetta by Drs. Brouardel and Cornil showed that the fatal issue was induced by perityphlitis and pericolitis, and that toward the end, a slight peritonitis had been lighted up. Profs. Charcot, Verneuil, Liouville, Guerdat and many other celebrities of the faculty were present, and signed the report. The brain was found to weigh 1,100 grammes.

DR. J. BURDON SANDERSON, editor of the "Handbook for the Pysiological Laboratory," has been appointed to the Waynflete Chair of Physiology at Oxford University, England.

IN the new German Parmacopœia, 360 articles have been struck out, and 48 added, a decrease of 312, the whole number being about 600. In the new edition of the U. S. Pharmacopœia 229 articles were cut out and 256 added, a net increase of 27, the whole number being 1,000.

CHARITY HOSPITAL, NEW YORK.—At the annual meeting of the Medical Board of Charity Hospital, at the Academy of Medicine, the following officers were elected for the current year: President, Dr. John H. Ripley; Vice-President, Dr. T. F. Ferguson; Secretary, Dr. Edward S. Peck.

IN No. 17 of *Le Progres Medical*, is an interesting account of cerebrospinal meningitis, occurring in a young woman toward the close of pregnancy. Her child developed the disease two hours after birth, and death rapidly ensued. This goes to show that the disease is properly classified, when placed under the head of miasmatic-contagious diseases.

COCKROACH TEA.—It is claimed that a decoction of female cockroaches (with the addition of a little brandy) is often used in practice, especially in the South, though no mention is made of it by prescriber or compounder, for obvious reasons. It is used as a diuretic in Russia, and also in certain forms of Bright's disease. Its properties are said to resemble those of cantharides.

THE IRISH COLLEGE OF PHYSICIANS has followed in the steps of the English college in adopting the following resolution: "That the advertisement of medical books in other than medical publications, and the giving by any of the licentiates, members and fellows of the college, whether for publication or not, laudatory certificates of medicinal or other preparations, or medical or surgical appliances, is misleading to the public, derogatory to the dignity of the profession, and is censurable by the college."

The proposed journal of the American Medical Association may now be regarded as a certainty. A sufficient number of subscriptions are assured to justify the Board of Trustees in taking steps for the publication of the first number on July 1, 1883.

New York Ophthalmological Society.—At the annual meeting, held January 8, 1883, the following officers were elected: President, George R. Cutter, M. D.; Vice-President, David Webster, M.D.; Secretary and Treasurer, Jas. L. Minor, M.D.

Local Anæsthesia.—Some experiments made in Germany in the production of local anæsthesia show that if the hand be immersed for a short time in ice water severe pain is caused, but no pain is produced on immersing the hand in cold alcohol of five degrees Cent. Glycerine was found to possess a similar property. Ether excited pain and quicksilver more acute pain still, causing the speedy withdrawal of the finger when plunged into this liquid at a temperature of three degrees. It was ascertained that, on the finger being held for a long time in alcohol having a temperature of five degrees Cent., no pain was experienced, and, although the finger distinctly perceived the faintest touch, sharp pricks gave no pain. This seemed to show that the application of cold alcohol has the effect of depriving the part of the special sensibility of pain, without, however, impairing the delicacy of the general tactile sensation which resides in the superficial integument.

St. Louis Dentists.—The regular meetings of the St. Louis Dental Society are held on the first Tuesday of each month. At the annual meeting, held Tuesday, January 2, 1883, the following were elected officers for the ensuing year: Dr. John G. Harper, President; Dr. J. B. Newby, Vice-President; Dr. A. H. Fuller, Corresponding Secretary; Dr. J. W. Whipple, Recording Secretary; Dr. Geo. A. Bowman, Treasurer.

The next meeting of the Brooklyn Dental Society will be held at the residence of Dr. Thos. Frey, No. 18 Clinton street, on Monday evening, February 12, 1883.

Hospital for Contagious Diseases.—The Board of Estimate and Apportionment has appropriated $50,000 for the erection of a hospital for children suffering from contagious diseases.

THE New York Society of Medical Jurisprudence will hold its second regular meeting on Thursday evening, February 8, 1883, at eight o'clock, at the Academy of Medicine, 12 West 31st street. Order—"Intellectual Monomania with Depression in its Medico-Legal Relations," as illustrated by the case of Duburque, the 14th Street assassin. By Prof. Wm. A. Hammond, M.D.

